# A Bitter-Sweet Story: Unraveling the Genes Involved in Quinolizidine Alkaloid Synthesis in *Lupinus albus*

**DOI:** 10.3389/fpls.2021.795091

**Published:** 2022-01-26

**Authors:** Claudia E. Osorio, Bradley J. Till

**Affiliations:** ^1^Instituto de Investigaciones Agropecuarias, INIA Carillanca, Temuco, Chile; ^2^Veterinary Genetics Laboratory, University of California, Davis, Davis, CA, United States

**Keywords:** alkaloids, quinolizidine alkaloids, white lupin, *Lupinus albus*, *pauper*

## Abstract

Alkaloids are part of a structurally diverse group of over 21,000 cyclic nitrogen-containing secondary metabolites that are found in over 20% of plant species. *Lupinus albus* are naturally containing quinolizidine alkaloid (QA) legumes, with wild accessions containing up to 11% of QA in seeds. Notwithstanding their clear advantages as a natural protecting system, lupin-breeding programs have selected against QA content without proper understanding of quinolizidine alkaloid biosynthetic pathway. This review summarizes the current status in this field, with focus on the utilization of natural mutations such as the one contained in *pauper* locus, and more recently the development of molecular markers, which along with the advent of sequencing technology, have facilitated the identification of candidate genes located in the *pauper* region. New insights for future research are provided, including the utilization of differentially expressed genes located on the *pauper locus*, as candidates for genome editing. Identification of the main genes involved in the biosynthesis of QA will enable precision breeding of low-alkaloid, high nutrition white lupin. This is important as plant based high quality protein for food and feed is an essential for sustainable agricultural productivity.

## Introduction

Lupinus, part of the Fabaceae family, is a genus of plants widely distributed around the world (Clements et al., 2008). Different cultivated species are present in the Old World (*L. albus, L. angustifolius*, and *L. luteus*) as well as the New World (*L. mutabilis*) (Petterson and Mackintosh, 1994; Peterson, 2000). Despite the phenotypic differences due to environmental conditions, they are all characterized by high level of proteins, being used as human food or animal feed (Petterson and Mackintosh, 1994; Abraham et al., 2019; Sońta and Rekiel, 2019). The production of lupin seeds as an agricultural product occurs mainly in Australia but also it has an important role in agricultural productive system in parts of Europe, Africa, and South America (FAO, 2021). During 2019, the largest lupin producers were Australia (474,629 t), Russian Federation (103,792 t) and Poland (261,500 t) (FAO, 2021).

White lupin (*Lupinus albus L*.) is a legume from the Mediterranean region; its center of origin is believed to be southern Greece and Western Turkey, where its cultivation started 4,000 years ago and wild landraces still persist (Gladstones et al., 1998). White lupin (WL) is recognized as an exceptional source of protein (between 30 and 40% of the whole seed dry matter) (Bähr et al., 2014) with an adequate balance of essential amino acids, as well as an adequate protein efficiency ratio (Sujak et al., 2006). During recent years, white lupin has gained attention for human consumption because of its levels of tocopherols, having the lowest glycemic index among consumed grains, high dietary fiber content and the absence of immunogenic epitopes causing celiac disease (Boschin et al., 2008; Boschin and Arnoldi, 2011; Fontanari et al., 2012; Bähr et al., 2014). It is also a crop with low need for phosphate fertilizers due to the presence of specialized cluster root structures and the capacity to releases phosphorus (P) from its insoluble form because of the ability to mobilize carboxylates (Lucas et al., 2015; Xu et al., 2020). Further, as a legume, application of nitrogen fertilizers can be avoided if an adequate symbiosis with *Rhyzobium* bacteria is achieved, decreasing therefore the environmental impact due to its cultivation (Pueyo et al., 2021).

However, the presence of secondary metabolites, which act as anti-nutritional compounds, in white lupin seeds limits its use. *Lupinus albus* wild varieties and landraces can accumulate up to 11% of their dry weight in the form of alkaloids, most of them belonging to the family of quinolizidine alkaloids (QA) (Rybiński et al., 2018). QA are notoriously bitter and toxic to both humans and farm animals, displaying both teratogenic and anti-cholinergic effects (Wink and Schimmer, 1999; Lourenço et al., 2002). Accordingly, traditional consumption of lupin grain involves a debittering process, which also removes a large proportion of nutrients such as soluble proteins, minerals, flavonoids, monosaccharides and sucrose from the seeds (Erbas, 2010). Despite WL importance and potential as a protein source, scarce knowledge has been generated about quinolizidine alkaloid synthesis. Cultivated WL relies on the incorporation of a natural occurring mutation at pauper locus, which decreases QA levels below the 0.02% threshold established as safe for consumption as food and feed, however, fundamental knowledge is required to maintain a reduction of QA within breeding programs.

The central objective of this review is to summarize the available resources to understand the biosynthesis of QA in *L. albus.* Identification and characterization of the genes responsible for QA biosynthesis are a challenge which breeders must pursue in order to manipulate the total amount and type present in any given genotype or commercial variety, while controlling the amount and type of QA and maintaining lupin high nutritional value. From an environmental point of view, breeding efforts could lead to optimizing selection for higher alkaloid content in the leaves (and thus resistance to pathogens and insects), while at the same time selecting for low QA in the seed (Gladstones et al., 1998). QA synthesis knowledge will provide the opportunity to ensure safe levels for human and animal consumption as well as optimal pest control, increasing yield, and decreasing the use of agrochemicals (Vishnyakova et al., 2020).

## Quinolizidine Alkaloid Biosynthetic Pathway

Alkaloids are part of a structurally diverse group of over 21,000 cyclic nitrogen-containing secondary metabolites (Wink, 2013) that are found in over 20% of plant species (Croteau et al., 2000; De Luca and St. Pierre, 2000; Bunsupa et al., 2012b). QA are secondary metabolites that occur mostly within the Leguminosae family, but are also present in other taxa (Ohmiya et al., 1995). In lupin species, QA distinctive structure is a quinolizidine ring, which can be grouped mainly into bicyclic alkaloids, such as lupinine and its derivatives, and tetracyclic alkaloids, represented by sparteine, lupanine and hydroxilupanine (Wink, 1987). Examples exist of distinctive QA expression in different species. Isolupanine and angustifoline accumulate to high levels in *L. angustifolius* (narrow-leafed lupin). Albinine and multiflorine accumulate to high levels in *L. albus* and lupinine in *L. luteus* (Święcicki et al., 2019). QA vary in their toxicity and their deterrence against insect pests and mammals. Sparteine and lupanine appear to be the two most toxic QA to humans and laboratory animals (Allen, 1998; Petterson, 1998), with lupanine having the greatest impact on aphid survival, followed by sparteine, lupinine, 13α-hydroxylupanine and angustifoline having a moderate impact (Ridsdill-Smith et al., 2004; Philippi et al., 2015). QA toxicity against larvae from different species as well as acaricidal effect has also been reported (Hassan et al., 2019; Elma et al., 2021).

The synthesis of QA occurs through the cyclization of cadaverine, due to a L-lysine decarboxylation catalyzed by a lysine decarboxylase L/ODC (La-L/ODC) (Saito and Murakoshi, 1995; Bunsupa et al., 2012a). Cadaverine is then oxidized by a copper amine oxidase (CuAO) (Yang et al., 2017) to yield 5-aminopentanal and spontaneously cyclized to 1-piperideine Schiff base, which is a universal intermediate for the production of various Lys-derived alkaloids (Bunsupa et al., 2012b). It has been suggested that in addition to these reactions, a series of aldol-type reactions, hydrolysis, oxidative deamination and coupling gives rise to the major structural QAs [e.g., lupanine and others; (Dewick, 2002)]. The diiminium cation was proposed as an intermediate product in to yield tetracyclic alkaloids [lupanine, multiflorine, and sparteine (Fraser and Robins, 1984)]. These QA are final products, but also, can be further modified by dehydrogenation, oxygenation, hydroxylation, glycosylation, acetylation or esterification to form a wide variety of structurally related QAs (Wink and Hartmann, 1982; Saito et al., 1992, 1993; Ohmiya et al., 1995; Saito and Murakoshi, 1995; Bunsupa et al., 2012b; Boschin and Resta, 2013). Continued research will likely add more detail to the QA pathway. For example, the observation that QA can be found in high enantiomeric excess has led to the proposal that stereoselective enzyme catalysis may be involved in the QA pathway (Lichman, 2021).

## Genes Involved in Quinolizidine Alkaloid Biosynthesis in *Lupinus*

Identification of genes involved in the QA biosynthesis has been partially achieved by identifying homologous genes in other species expressing QA, as in the case of *Lupinus angustifolius La-L/ODC* gene, which was identified as a homolog of L/*ODC* expressed in the distantly related species *S. flavescens*, *E. koreensis*, *T. chinensis*, and *B. australis* (Bunsupa et al., 2012a). Genes encoding acyltransferase were described in *L. albus* and *L. angustifolius* (*LaHMT/HLT* and *LaAT*, *respectively*), but proof of the formation of acetylated products (13α-hydroxylupanine and 13α-hydroxymultiflorine), was only achieved for *L. albus HMT/HLT* (Saito and Murakoshi, 1995; Okada et al., 2005; Bunsupa et al., 2011).

Transcriptome experiments in different tissues of *L. angustifolius* lead to the identification of a copper amine oxidase, LaCAO (Yang et al., 2017), with cadaverine as substrate, catalyzing its transformation into 5-aminopentanal, which is then spontaneously cyclized to 1-piperideine (Yang et al., 2017). In a previous report, Okada et al. (2005) cloned and characterized an *O*-tigloytransferase from WL, an enzyme involved in the final steps of QA biosynthesis. Recently, it was proven for *Lupinus angustifolius*, that *RAP2-7* is a putative regulatory gene of QA biosynthesis/accumulation in aerial tissues (Kroc et al., 2019), with a S196R substitution being responsible for the bitter/sweet phenotype (Czepiel et al., 2021), but, however, additional studies are needed to determine the mechanism and effect on QA expression, and its role in different lupin species. In an effort to identify the missing enzymes of the QA pathway, the study of the existence of common enzymes between nicotine synthesis (monoterpene indole alkaloids, MIA; benzylisoquinole alkaloids, BIA) and lupins QA has been proposed (Frick et al., 2017). Many of these enzymes (methyltransferases, decarboxylases, oxidases, acyltransferases, cytochromes-P450, oxidoreductases, demethylases, reductases, hydroxylases, and coupling enzymes) and their encoding genes have been identified in *N. tabacum, C. roseus, C. japonica*, and *P. somniferum* (Bird et al., 2003; Dewey and Xie, 2013; Hagel and Facchini, 2013; Kilgore and Kutchan, 2016; Pan et al., 2016; Thamm et al., 2016) and it is expected that they play a role in lupins QA biosynthesis (Frick et al., 2017). *Sophora flavescens* transcriptome analysis had also identified several genes co-expressed, such as a putative *S. flavescens* L/ODC and candidate genes clustered into the same clade as L/ODC (major latex-like protein (MLP-like), a cP450, and a ripening related protein), but their function remains unknown (Han et al., 2015). Berberine bridge and berberine bridge-like enzymes catalyze oxidative reactions for the biosynthesis of BIAs (Facchini et al., 1996; Samanani et al., 2004; Kajikawa et al., 2011), possibly having similar roles in QA biosynthesis. Cytocromes-P450 have a role in hydroxylation reactions, as well as other reactions in MIA and BIA biosynthesis (Pauli and Kutchan, 1998; Thamm et al., 2016). Recently, the existence of a high number of QA biosynthesis genes controlled by a regulatory agent localized at *iucundus* locus in NLL was reported, which supports the idea that ethylene responsive transcription factor *RAP2-7* gene may control low-alkaloid phenotype in NLL, acting as a promoter of the expression of biosynthesis genes (Plewiński et al., 2019; Czepiel et al., 2021).

In addition to QAs biosynthetic genes, major *loci* controlling QA expression have been described in lupins. Cultivated lupins display lower alkaloid content than landraces, due to incorporation of “sweet” domestication genes, which were generated by natural mutation (Lin et al., 2009). Most of these mutations are recessive, such as *iucundus, esculentus*, and *depressus* in NLL, *amoenus, dulcis*, and *liber* in *L. luteus* (Lin et al., 2009). In *L. albus*, several *loci* have been reported to produce low alkaloid genotypes, with the *pauper* locus being the most effective and used worldwide in breeding programs (Gladstones, 1974; Harrison and Willliams, 1982). In *L. mutabilis*, the low alkaloid phenotype is controlled by several alleles (Clements et al., 2008). It is worth highlighting that none of the mutations identified in lupin completely eliminate QAs (Harrison and Willliams, 1982).

## *Pauper* Locus

Construction of low-density linkage maps allowed identification of genomic regions involved in alkaloid biosynthesis in white lupin (Phan et al., 2007; Croxford et al., 2008; Vipin et al., 2013). There has also been development of molecular markers to identify QTLs responsible for low alkaloid content linked to these recessive loci (Yang et al., 2010; Raman et al., 2014). The development and mass use of GBS technology as a tool for breeders (Elshire et al., 2011; Annicchiarico et al., 2017), enabled progress to identify causative genes for low QA content in *L. albus*. High-density consensus maps for comparisons between *L. angustifolious* and *L. albus*, had led to the hypothesis that the *iucundus* locus responsible for low alkaloid content in NLL differs by function from *pauper* in WL (Książkiewicz et al., 2017).

Worldwide, *L. albus* breeding programs have relied mainly on the effect of *pauper locus* to produce sweet varieties, for both food and feed. Besides its importance, little is known about the *pauper* locus gene(s) with respect to their effect on alkaloid content. Earlier studies identified at least two different alleles for *pauper locus*, controlling total alkaloid content (Harrison and Willliams, 1982). The action of this recessive locus was suggested as a reduction of a common alkaloid substrate, which seems to be the ubiquitous for most lupin low alkaloid genotypes, without affecting intermediate substrates at late biosynthetic stages, when chemical differences among alkaloids are being finally specified (Harrison and Willliams, 1982).

Genetic and comparative map of *L. albus*, based on a RIL population developed from Kiev (Ukrainian cv, sweet, early flowering, anthracnose susceptible) and P27174 (Ethiopian landrace, bitter, late flowering, anthracnose resistant), allowed the discovery of 220 amplified fragment length polymorphisms and 105 gene-based markers, enabling for the first time mapping of the alkaloid locus, with flanking markers located in a region within 20 cM in both directions (Phan et al., 2007). Later, with the development of Pauper M1, a molecular marker more closely linked to the pauper locus (1.4 cM), allowed discrimination of low alkaloid content genotypes with efficiencies restricted to ∼95% for bitter and 91% for sweet non-*pauper* lines. Implementing PauperM1 required the use of sequencing gels and radioisotope primer labeling for the correct determination of alleles, which made its application restricted to authorized facilities (Lin et al., 2009). With the aid of GBS, a high-density consensus linkage map of WL genome was constructed, integrating 453 published markers with 3,597 newly developed sequence-based markers, recovering a single linkage group per every chromosome (Książkiewicz et al., 2017). This map yielded several new markers co-segregating, or closely localized to the *pauper* locus than the Pauper M1 (Książkiewicz et al., 2017). In an effort to improve Pauper M1 efficacy, CAPS markers were developed (using two identified SNPs), which were substrates for restriction enzymes, *Hha*I for the bitter allele, and *Hin*fI for the sweet allele (Rychel and Książkiewicz, 2019). One of these, LAGI01_35805_F1_R1 homologous to *LaAT* (AB581532.1), different than *pauper* locus gene, showed higher efficacy than Pauper M1 (Rychel and Książkiewicz, 2019).

Recently a high quality reference *L. albus* genome allowed the study of the *pauper* genomic region, identifying several candidate genes on Chr18. This research demonstrate the existence of a gene cluster in the *pauper* locus, which comprises a 958 kb region and contains 66 genes, amongst which several are strong candidates genes encoding enzymatic activities, such as cinnamoyl-CoA reductase and acyltransferases (Hufnagel et al., 2020a). In addition to the reference genome, transcriptomic data from different organs, resequencing of 15 varieties and a pangenome dataset provide tools for further exploration of the genomics of alkaloid content^1^ (Hufnagel et al., 2020b) and the complex role of the pauper locus. Analysis of the pauper marker associated with low QA was carried out in a landrace, breeding lines and cultivars of *L. albus* and QA was measured by UHPLC-HRMS. Interestingly while the marker did associate with low QA and was absent in many high QA samples, there were notable exceptions where the marker was found in high QA sample (Zafeiriou et al., 2021). Thus additional genes and regulatory elements may be important in reaching breeding objectives.

## Prospective Techniques to Unravel Biosynthesis Alkaloid Genes in White Lupin

The main drawback to study candidate genes and their function in WL is the recalcitrant nature of this legume to tissue culture (Nguyen et al., 2016; Aslam et al., 2020). Several attempts have been undertaken to develop *in vitro* regeneration tissue culture systems, but with limited success, which today represent a challenge in WL breeding programs (Bayliss et al., 2002; Uhde-Stone et al., 2005; Surma et al., 2013; Raza et al., 2017; Che et al., 2019; Aslam et al., 2020). It is possible, as in monocot species, that transformation is highly cultivar dependent, therefore a genetic screen for transformation aptitude in WL collections may produce a genotype amenable to transformation and thus genome editing. But, in the meantime, to overcome this situation, different reverse genetics methodologies (Till et al., 2007), such as random mutagenesis and virus induced gene silencing (VIGS) may be used to probe gene function (Gupta et al., 2013).

During the last years, the use of transient expression has facilitated gene-discovery by utilization of VIGS (Gupta et al., 2013). VIGS is an effective tool to characterize functions of candidate genes using post-transcriptional gene silencing (PTGS), which is extensively used for gene knockdowns in plants (Liu et al., 2016; Wang et al., 2016; Zhang et al., 2016). However, VIGS can also be applied as a forward genetics technique to study gene function by using cDNA libraries (Kilgore and Kutchan, 2016; Thamm et al., 2016). In white lupin, VIGS using peanut stunt virus proved to be effective tool to silence the Phytoene desaturase gene (LaPDS) (Yamagishi et al., 2015), opening possibilities for utilization of this technique to elucidate genes participating in secondary metabolite synthesis such as QA.

Advances in CRISPR-Cas technology allow fine-tuning of gene-activity and the generation larger chromosomal variation, providing a broad toolkit for gene-function analysis (Jinek et al., 2012; Cong et al., 2013; Jung and Till, 2021). Nevertheless, optimization of CRISPR technology is needed to accommodate the tissue and transformation delivery method (Char et al., 2017). Recent developments of new transformation techniques based on the utilization of functionalized nanoparticles to deliver DNA, has been proven successful in species such as cotton, sunflower and lily (Zhao et al., 2017), opening the possibilities to bypass *in vitro* regeneration in legume species, such as lupin. With the aid of magnetic fields, nanoparticles can efficiently deliver CRISPR vectors through pores present in the pollen grains, producing transformed pollen which is then used to pollinate emasculated flowers, resulting in transformed seeds (Zhao et al., 2017). The utilization of functionalized magnetic particles to deliver DNA into pollen grains, and to accelerate selection of desired individuals using speed-breeding (Watson et al., 2018; Lew et al., 2020) increases transformation efficiency and bypasses tissue culture procedures to generate plants from transformed seeds within a short period of time, broadening the possibilities for WL utilization as mayor knowledge on QA synthesis is achieved. Continued advances in nanoparticle technologies may make CRISPR-Cas approaches amenable in many recalcitrant species (Ma et al., 2021).

## Summary

**FIGURE 1:**
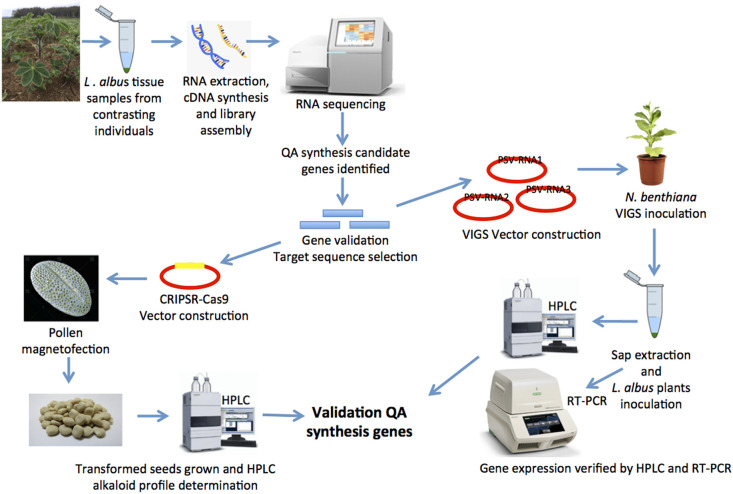
A strategy to unravel quinolizidine alkaloid (QA) synthesis genes using virus induced gene silencing and genome editing, for functional genomics and crop improvement in *Lupinus albus.*

Quinolizidine alkaloid synthesis has an important number of unresolved questions, which hinders breeding efforts worldwide in a crop with high nutritional quality such as WL. Utilization of techniques to study and manipulate genes involved in alkaloid synthesis in *L. albus* will contribute to a better understanding of the accumulation of secondary metabolites in lupin seed (Figure 1), contributing to the development of environmentally friendly and sustainable sources of plant protein, which are expected to be a key component of conscientious population growth. Continued efforts in white lupin breeding, leveraging knowledge gained of the genetics of QA synthesis, can have an important role in human nutrition and well being.

## Author Contributions

CO and BT wrote, edited and reviewed the manuscript, and approved the submitted version.

## Conflict of Interest

The authors declare that the research was conducted in the absence of any commercial or financial relationships that could be construed as a potential conflict of interest.

## Publisher’s Note

All claims expressed in this article are solely those of the authors and do not necessarily represent those of their affiliated organizations, or those of the publisher, the editors and the reviewers. Any product that may be evaluated in this article, or claim that may be made by its manufacturer, is not guaranteed or endorsed by the publisher.

## Footnotes

1
https://www.whitelupin.fr/

## References

[B1] Abraham E. M. Ganopoulos I. Madesis P. Mavromatis A. Mylona P. Nianiou-Obeidat I. (2019). The use of lupin as a source of protein in animal feeding: genomic tools and breeding approaches. *Int. J. Mol. Sci.* 20:851. 10.3390/ijms20040851 PMC6413129 30781397

[B2] Allen J. G. (1998). “Toxins and lupinosis” in *Lupins as a Crop Plant Biology, Production and Utilization.* eds Gladstones J. S. Atkins C. A. Hamblin J. (Cambridge: CAB International, University Press). 411–428.

[B3] Annicchiarico P. Nazzicari N. Wei Y. Pecetti L. Brummer E. C. (2017). Genotyping-by-Sequencing and its exploitation for forage and cool-season grain legume breeding. *Front. Plant Sci.* 8:679. 10.3389/fpls.2017.00679 28536584 PMC5423274

[B4] Aslam M. M. Karanja J. K. Zhang Q. Lin H. Xia T. Akhtar K. (2020). In Vitro Regeneration Potential of White Lupin (Lupinus albus) from Cotyledonary Nodes. *Plants* 9:318. 10.3390/plants9030318 32138269 PMC7154923

[B5] Bähr M. Fechner A. Hasenkopf K. Mittermaier S. Jahreis G. (2014). Chemical composition of dehulled seeds of selected lupin cultivars in comparison to pea and soya bean. *LWT Food Sci. Technol.* 59 587–590. 10.1016/j.lwt.2014.05.026

[B6] Bayliss K. Wroth J. Cowling W. (2002). *Pro-embryos of Lupinus Albus Produced from Isolated Microspore Culture.* Australian: The Australian Plant Breeding Association Inc., 57–61.

[B7] Bird D. A. Franceschi V. R. Facchini P. J. (2003). A tale of three cell types: alkaloid biosynthesis is localized to sieve elements in opium poppy. *Plant Cell.* 15 2626–2635. 10.1105/tpc.015396 14508000 PMC280566

[B8] Boschin G. Arnoldi A. (2011). Legumes are valuable sources of tocopherols. *Food Chem.* 127 1199–1203. 10.1016/j.foodchem.2011.01.124 25214114

[B9] Boschin G. D’Agostina A. Annicchiarico P. Arnoldi A. (2008). Effect of genotype and environment on fatty acid composition of Lupinus albus L. seed. *Food Chem.* 108 600–606. 10.1016/j.foodchem.2007.11.016 26059138

[B10] Boschin G. Resta D. (2013). “Alkaloids Derived from Lysine: quinolizidine (a Focus on Lupin Alkaloids)” in *Natural Products: phytochemistry, Botany and Metabolism of Alkaloids, Phenolics and Terpenes.* eds Ramawat G. K. Mérillon J.-M. (Berlin: Springer). 381–403. 10.1007/978-3-642-22144-6_11

[B11] Bunsupa S. Katayama K. Ikeura E. Oikawa A. Toyooka K. Saito K. (2012a). Lysine decarboxylase catalyzes the first step of quinolizidine alkaloid biosynthesis and coevolved with alkaloid production in leguminosae. *Plant Cell* 24 1202–1216. 10.1105/tpc.112.095885 22415272 PMC3336119

[B12] Bunsupa S. Yamazaki M. Saito K. (2012b). Quinolizidine alkaloid biosynthesis: recent advances and future prospects. *Front. Plant Sci.* 3:239. 10.3389/fpls.2012.00239 23112802 PMC3481059

[B13] Bunsupa S. Okada T. Saito K. Yamazaki M. (2011). An acyltransferase-like gene obtained by differential gene expression profiles of quinolizidine alkaloid-producing and nonproducing cultivars of Lupinus angustifolius. *Plant Biotechnol.* 28 89–94. 10.5511/plantbiotechnology.10.1109b

[B14] Char S. N. Neelakandan A. K. Nahampun H. Frame B. Main M. Spalding M. H. (2017). An Agrobacterium-delivered CRISPR/Cas9 system for high-frequency targeted mutagenesis in maize. *Plant Biotechnol. J.* 15 257–268. 10.1111/pbi.12611 27510362 PMC5259581

[B15] Che P. Chang S. Simon M. K. Zhang Z. Shaharyar A. Ourada J. (2019). Developing a rapid and highly efficient cowpea regeneration and transformation system using embryonic axis explants. *Biorxiv* 738971. 10.1101/738971 PMC8252785 33595147

[B16] Clements J. Prilyuk L. Quealy J. Francis G. (2008). “Interspecific crossing among the New World lupin species for Lupinus mutabilis crop improvement” in *Lupins for Health and Wealth Proceedings of the 12th International Lupin Conference.* eds Palta J. A. Berger J. B. (Western Australia: International Lupin Association). 14.

[B17] Cong L. Ran F. A. Cox D. Lin S. Barretto R. Habib N. (2013). Multiplex genome engineering using CRISPR/Cas systems. *Science* 339 819–823. 23287718 10.1126/science.1231143 PMC3795411

[B18] Croteau R. Kutchan T. M. Lewis N. G. (2000). Natural products (secondary metabolites). *Biochem. Mol. Biol. Plants* 24 1250–1319. 10.1128/mSystems.00186-17 29556536 PMC5850076

[B19] Croxford A. E. Rogers T. Caligari P. D. Wilkinson M. J. (2008). High-resolution melt analysis to identify and map sequence-tagged site anchor points onto linkage maps: a white lupin (Lupinus albus) map as an exemplar. *New Phytol.* 180 594–607. 10.1111/j.1469-8137.2008.02588.x 18684160

[B20] Czepiel K. Krajewski P. Wilczura P. Bielecka P. Święcicki W. Kroc M. (2021). Expression Profiles of Alkaloid-Related Genes across the Organs of Narrow-Leafed Lupin (Lupinus angustifolius L.) and in Response to Anthracnose Infection. *Int. J. Mol. Sci.* 22:2676. 10.3390/ijms22052676 33800929 PMC7962062

[B21] De Luca V. St. Pierre B. (2000). The cell and developmental biology of alkaloid biosynthesis. *Trends Plant Sci.* 5 168–173. 10.1016/s1360-1385(00)01575-2 10740298

[B22] Dewey R. E. Xie J. (2013). Molecular genetics of alkaloid biosynthesis in Nicotiana tabacum. *Phytochemistry* 94 10–27. 10.1016/j.phytochem.2013.06.002 23953973

[B23] Dewick P. M. (2002). *Medicinal Natural Products: a Biosynthetic Approach.* United States: John Wiley & Sons.

[B24] Elma F. N. Çetín H. Yorgancilar M. Acar R. (2021). Detection of Metabolite Content in Local Bitter White Lupin Seeds (Lupinus albus L.) and Acaricidal and Insecticidal Effect of its Seed Extract. *Tarım Biliml. Derg.* 27 407–413.

[B25] Elshire R. J. Glaubitz J. C. Sun Q. Poland J. A. Kawamoto K. Buckler E. S. (2011). A robust, simple genotyping-by-sequencing (GBS) approach for high diversity species. *PLoS One* 6:e19379. 10.1371/journal.pone.0019379 21573248 PMC3087801

[B26] Erbas M. (2010). The effects of different debittering methods on the production of lupin bean snack from bitter Lupinus albus L. seeds. *J. Food Qual.* 33 742–757. 10.1111/j.1745-4557.2010.00347.x

[B27] Facchini P. J. Penzes C. Johnson A. G. Bull D. (1996). Molecular characterization of berberine bridge enzyme genes from opium poppy. *Plant Physiol.* 112 1669–1677. 10.1104/pp.112.4.1669 8972604 PMC158100

[B28] FAO (2021). *FAOSTAT. Crops and Livestock Products.* Available online at: https://www.fao.org/faostat/en/#data/QCL (accessed January 07, 2022).

[B29] Fontanari G. G. Batistuti J. P. da Cruz R. J. Saldiva P. H. N. Aręas J. A. G. (2012). Cholesterol-lowering effect of whole lupin (Lupinus albus) seed and its protein isolate. *Food Chem.* 132 1521–1526. 10.1016/j.foodchem.2011.11.145 29243644

[B30] Fraser A. M. Robins D. J. (1984). Incorporation of chiral [1-2 H] cadaverines into the quinolizidine alkaloids sparteine, lupanine, and angustifoline. *J. Chem. Soc. Chem. Commun.* 1477–1479. 10.1039/c39840001477

[B31] Frick K. M. Kamphuis L. G. Siddique K. H. Singh K. B. Foley R. C. (2017). Quinolizidine alkaloid biosynthesis in lupins and prospects for grain quality improvement. *Front. Plant Sci.* 8:87. 10.3389/fpls.2017.00087 28197163 PMC5281559

[B32] Gladstones J. S. (1974). *Lupins of the Mediterranean region and Africa.* Perth: Department of Agriculture of Western Australia.

[B33] Gladstones J. S. Atkins C. Hamblin J. (1998). *Lupins as Crop Plants: biology, Production and Utilization.* UK: CAB international.

[B34] Gupta B. Saha J. Sengupta A. Gupta K. (2013). Recent advances on Virus Induced Gene silencing (VIGS): plant functional genomics. *J. Plant Biochem. Physiol.* 1:e116. 10.4172/2329-9029.1000e116

[B35] Hagel J. M. Facchini P. J. (2013). Benzylisoquinoline alkaloid metabolism: a century of discovery and a brave new world. *Plant Cell Physiol.* 54 647–672. 10.1093/pcp/pct020 23385146

[B36] Han R. Takahashi H. Nakamura M. Bunsupa S. Yoshimoto N. Yamamoto H. (2015). Transcriptome analysis of nine tissues to discover genes involved in the biosynthesis of active ingredients in Sophora flavescens. *Biol. Pharmaceut. Bull.* 38 876–883. 10.1248/bpb.b14-00834 26027827

[B37] Harrison J. Willliams W. (1982). Genetical control of alkaloids in Lupinus albus. *Euphytica* 31 357–364. 10.1007/bf00021651

[B38] Hassan M. E. Aly N. E.-D. S. Mikhail M. W. (2019). Larvicidal effect of alkaloids extracted from bitter lupin seeds against mosquitoes (Culex pipiens), flies (Musca domestica) and fleas (Xenopsylla cheopis) under laboratory conditions in Egipt. *J. Egypt. Soc. Parasitol.* 49 455–464. 10.21608/jesp.2019.68192

[B39] Hufnagel B. Marques A. Soriano A. Marquès L. Divol F. Doumas P. (2020a). High-quality genome sequence of white lupin provides insight into soil exploration and seed quality. *Nat. Commun.* 11 1–12. 10.1038/s41467-019-14197-9 31980615 PMC6981116

[B40] Hufnagel B. Soriano A. Taylor J. Divol F. Kroc M. Sanders H. (2020b). Pangenome of white lupin provides insights into the diversity of the species. *Biorxiv* 10.1111/pbi.13678 34346542 PMC8633493

[B41] Jinek M. Chylinski K. Fonfara I. Hauer M. Doudna J. A. Charpentier E. (2012). A programmable dual-RNA–guided DNA endonuclease in adaptive bacterial immunity. *Science* 337 816–821. 10.1126/science.1225829 22745249 PMC6286148

[B42] Jung C. Till B. (2021). Mutagenesis and genome editing in crop improvement: perspectives for the global regulatory landscape. *Trends Plant Sci.* 26 1258–1269. 10.1016/j.tplants.2021.08.002 34465535

[B43] Kajikawa M. Shoji T. Kato A. Hashimoto T. (2011). Vacuole-localized berberine bridge enzyme-like proteins are required for a late step of nicotine biosynthesis in tobacco. *Plant Physiol.* 155 2010–2022. 10.1104/pp.110.170878 21343426 PMC3091092

[B44] Kilgore M. B. Kutchan T. M. (2016). The Amaryllidaceae alkaloids: biosynthesis and methods for enzyme discovery. *Phytochem. Rev.* 15 317–337. 10.1007/s11101-015-9451-z 27340382 PMC4914137

[B45] Kroc M. Koczyk G. Kamel K. A. Czepiel K. Fedorowicz-Strońska O. Krajewski P. (2019). Transcriptome-derived investigation of biosynthesis of quinolizidine alkaloids in narrow-leafed lupin (Lupinus angustifolius L.) highlights candidate genes linked to iucundus locus. *Sci. Rep.* 9 1–13. 10.1038/s41598-018-37701-5 30783128 PMC6381137

[B46] Książkiewicz M. Nazzicari N. Nelson M. N. Renshaw D. Rychel S. Ferrari B. (2017). A high-density consensus linkage map of white lupin highlights synteny with narrow-leafed lupin and provides markers tagging key agronomic traits. *Sci. Rep.* 7 1–15. 10.1038/s41598-017-15625-w 29127429 PMC5681670

[B47] Lew T. T. S. Park M. Wang Y. Gordiichuk P. Yeap W.-C. Mohd Rais S. K. (2020). Nanocarriers for transgene expression in pollen as a plant biotechnology tool. *ACS Mater. Lett.* 2 1057–1066. 10.1021/acsmaterialslett.0c00247

[B48] Lichman B. R. (2021). The scaffold-forming steps of plant alkaloid biosynthesis. *Nat. Prod. Rep.* 38 103–129. 10.1039/d0np00031k 32745157

[B49] Lin R. Renshaw D. Luckett D. Clements J. Yan G. Adhikari K. (2009). Development of a sequence-specific PCR marker linked to the gene “pauper” conferring low-alkaloids in white lupin (Lupinus albus L.) for marker assisted selection. *Mol. Breed.* 23 153–161. 10.1007/s11032-008-9222-2

[B50] Liu N. Xie K. Jia Q. Zhao J. Chen T. Li H. (2016). Foxtail mosaic virus-induced gene silencing in monocot plants. *Plant Physiol.* 171 1801–1807. 10.1104/pp.16.00010 27225900 PMC4936545

[B51] Lourenço A. Máximo P. Ferreira L. Pereira M. (2002). Indolizidine and quinolizidine alkaloids structure and bioactivity. *Stud. Nat. Prod. Chem.* 27 233–298. 10.1016/s1572-5995(02)80038-2

[B52] Lucas M. M. Stoddard F. Annicchiarico P. Frias J. Martinez-Villaluenga C. Sussmann D. (2015). The future of lupin as a protein crop in Europe. *Front. Plant Sci.* 6:705. 10.3389/fpls.2015.00705 26442020 PMC4561814

[B53] Ma K. Li W. Zhu G. Sun S. Chi H. Yin Y. (2021). Functionalized PDA/DEX-PEI@ HA nanoparticles combined with sleeping-beauty transposons for multistage targeted delivery of CRISPR/Cas9 gene. *Biomed. Pharmacother.* 142:112061. 10.1016/j.biopha.2021.112061 34449313

[B54] Nguyen A. H. Hodgson L. M. Erskine W. Barker S. J. (2016). An approach to overcoming regeneration recalcitrance in genetic transformation of lupins and other legumes. *Plant Cell Tissue Organ Cult.* 127 623–635. 10.1007/s11240-016-1087-1

[B55] Ohmiya S. Saito K. Murakoshi I. (1995). “Lupin alkaloids” in *The Alkaloids: chemistry and Pharmacology.* ed Cordell G. A. (San Diego: Academic Press). 1–114.

[B56] Okada T. Hirai M. Y. Suzuki H. Yamazaki M. Saito K. (2005). Molecular characterization of a novel quinolizidine alkaloid O-tigloyltransferase: cDNA cloning, catalytic activity of recombinant protein and expression analysis in Lupinus plants. *Plant Cell Physiol.* 46 233–244. 10.1093/pcp/pci021 15659437

[B57] Pan Q. Mustafa N. R. Tang K. Choi Y. H. Verpoorte R. (2016). Monoterpenoid indole alkaloids biosynthesis and its regulation in Catharanthus roseus: a literature review from genes to metabolites. *Phytochem. Rev.* 15 221–250. 10.1007/s00709-011-0291-4 21630129

[B58] Pauli H. H. Kutchan T. M. (1998). Molecular cloning and functional heterologous expression of two alleles encoding (S)-N-methylcoclaurine 3′-hydroxylase (CYP80B1), a new methyl jasmonate-inducible cytochrome P-450-dependent mono-oxygenase of benzylisoquinoline alkaloid biosynthesis. *Plant J.* 13 793–801. 10.1046/j.1365-313x.1998.00085.x 9681018

[B59] Peterson D. S. (2000). The use of Lupins in Feeding Systems. *Asian Austr. J. Anim. Sci.* 13 861–882. 10.5713/ajas.2000.861

[B60] Petterson D. (1998). “Composition and food uses of lupin” in *Lupins as Crop Plants: biology, Production, and Utilization.* eds Gladstones J. Atkins C. Wallingford H. (UK: CAB International). 353–384.

[B61] Petterson D. Mackintosh J. B. (1994). *The Chemical Composition and Nutritive Value of Australian Grain Legumes.* Canberra: Grains Research and Development Corp.

[B62] Phan H. T. Ellwood S. R. Adhikari K. Nelson M. N. Oliver R. P. (2007). The first genetic and comparative map of white lupin (Lupinus albus L.): identification of QTLs for anthracnose resistance and flowering time, and a locus for alkaloid content. *DNA Res.* 14 59–70. 10.1093/dnares/dsm009 17526914 PMC2779896

[B63] Philippi J. Schliephake E. Jürgens H. U. Jansen G. Ordon F. (2015). Feeding behavior of aphids on narrow-leafed lupin (L upinus angustifolius) genotypes varying in the content of quinolizidine alkaloids. *Entomol. Exp. Appl.* 156 37–51. 10.1111/eea.12313

[B64] Plewiński P. Książkiewicz M. Rychel-Bielska S. Rudy E. Wolko B. (2019). Candidate Domestication-Related Genes Revealed by Expression Quantitative Trait Loci Mapping of Narrow-Leafed Lupin (Lupinus angustifolius L.). *Int. J. Mol. Sci.* 20:5670. 10.3390/ijms20225670 31726789 PMC6888189

[B65] Pueyo J. J. Quińones M. A. Coba de la Peńa T. Fedorova E. E. Lucas M. M. (2021). Nitrogen and Phosphorus Interplay in Lupin Root Nodules and Cluster Roots. *Front. Plant Sci.* 12:644218. 10.3389/fpls.2021.644218 33747024 PMC7966414

[B66] Raman R. Cowley R. B. Raman H. Luckett D. J. (2014). Analyses using SSR and DArT molecular markers reveal that Ethiopian accessions of white lupin (Lupinus albus L.) represent a unique genepool. *Open J. Genet.* 04 87–98. 10.4236/ojgen.2014.42012

[B67] Raza G. Singh M. B. Bhalla P. L. (2017). In Vitro Plant Regeneration from Commercial Cultivars of Soybean. *Biomed. Res. Int.* 2017:7379693. 10.1155/2017/7379693 28691031 PMC5485301

[B68] Ridsdill-Smith T. Edwards O. Wang S. F. Ghisalberti E. Reidy-Crofts J. (2004). “Aphid responses to plant defensive compounds in lupins”in *Aphids in a New Millennium.* eds Simon J. C. Dedryver C. A. Rispe C. Hullé M. (France: INRA). 491–497.

[B69] Rybiński W. Kroc M. Święcicki W. Wilczura P. Kamel K. Barzyk P. (2018). *Preliminary Estimation of Variation of Alkaloids Content in White Lupin (Lupinus albus L.) Collection.* Germany: Springer. 131–136.

[B70] Rychel S. Książkiewicz M. (2019). Development of gene-based molecular markers tagging low alkaloid pauper locus in white lupin (Lupinus albus L.). *J. Appl. Genet.* 60 269–281. 10.1007/s13353-019-00508-9 31410824 PMC6803572

[B71] Saito K. Koike Y. Suzuki H. Murakoshi I. (1993). Biogenetic implication of lupin alkaloid biosynthesis in bitter and sweet forms of Lupinus luteus and L. albus. *Phytochemistry* 34 1041–1044. 10.1016/s0031-9422(00)90709-x

[B72] Saito K. Murakoshi I. (1995). Chemistry, biochemistry and chemotaxonomy of lupin alkaloids in the Leguminosae. *Stud. Nat. Prod. Chem.* 15 519–549. 10.1016/s1572-5995(06)80142-0

[B73] Saito K. Suzuki H. Takamatsu S. Murakoshi I. (1992). Acyltransferases for lupin alkaloids in Lupinus hirsutus. *Phytochemistry* 32 87–91.

[B74] Samanani N. Liscombe D. K. Facchini P. J. (2004). Molecular cloning and characterization of norcoclaurine synthase, an enzyme catalyzing the first committed step in benzylisoquinoline alkaloid biosynthesis. *Plant J.* 40 302–313. 10.1111/j.1365-313X.2004.02210.x 15447655

[B75] Sońta M. Rekiel A. (2019). Legumes—Use for nutritional and feeding purposes. *J. Elementol.* 25 835–849.

[B76] Sujak A. Kotlarz A. Strobel W. (2006). Compositional and nutritional evaluation of several lupin seeds. *Food Chem.* 98 711–719.

[B77] Surma M. Adamski T. Swiecicki W. Barzyk P. Kaczmarek Z. Kuczynska A. (2013). Preliminary results of in vitro culture of pea and lupin embryos for the reduction of generation cycles in single seed descent technique. *Acta Soc. Botanicor. Polon.* 82:3.

[B78] Święcicki W. Czepiel K. Wilczura P. Barzyk P. Kaczmarek Z. Kroc M. (2019). Chromatographic fingerprinting of the Old World lupins seed alkaloids: a supplemental tool in species discrimination. *Plants* 8:548. 10.3390/plants8120548 31783673 PMC6963311

[B79] Thamm A. M. Qu Y. De Luca V. (2016). Discovery and metabolic engineering of iridoid/secoiridoid and monoterpenoid indole alkaloid biosynthesis. *Phytochem. Rev.* 15 339–361. 10.1007/s11101-016-9468-y

[B80] Till B. J. Cooper J. Tai T. H. Colowit P. Greene E. A. Henikoff S. (2007). Discovery of chemically induced mutations in rice by TILLING. *BMC Plant Biol.* 7:19. 10.1186/1471-2229-7-19 17428339 PMC1858691

[B81] Uhde-Stone C. Liu J. Zinn K. E. Allan D. L. Vance C. P. (2005). Transgenic proteoid roots of white lupin: a vehicle for characterizing and silencing root genes involved in adaptation to P stress. *Plant J.* 44 840–853. 10.1111/j.1365-313X.2005.02573.x 16297074

[B82] Vipin C. A. Luckett D. J. Harper J. D. Ash G. J. Kilian A. Ellwood S. R. (2013). Construction of integrated linkage map of a recombinant inbred line population of white lupin (Lupinus albus L.). *Breed. Sci.* 63 292–300. 10.1270/jsbbs.63.292 24273424 PMC3770556

[B83] Vishnyakova M. Kushnareva A. Shelenga T. Egorova G. (2020). Alkaloids of narrow-leaved lupine as a factor determining alternative ways of the crop’s utilization and breeding. *Vavilov J. Genet. Breed.* 24:625. 10.18699/VJ20.656 33659848 PMC7716546

[B84] Wang R. Yang X. Wang N. Liu X. Nelson R. S. Li W. (2016). An efficient virus-induced gene silencing vector for maize functional genomics research. *Plant J.* 86 102–115. 10.1111/tpj.13142 26921244

[B85] Watson A. Ghosh S. Williams M. J. Cuddy W. S. Simmonds J. Rey M.-D. (2018). Speed breeding is a powerful tool to accelerate crop research and breeding. *Nat. Plants* 4 23–29. 10.1038/s41477-017-0083-8 29292376

[B86] Wink M. (1987). Physiology of the accumulation of secondary metabolites with special reference to alkaloids. *Cell Cult. Somat. Cell Genet. Plants* 4 17–42. 10.1016/b978-0-12-715004-8.50008-9

[B87] Wink M. (2013). Evolution of secondary metabolites in legumes (Fabaceae). *South Afr. J. Bot.* 89 164–175. 10.1016/j.sajb.2013.06.006

[B88] Wink M. Hartmann T. (1982). Localization of the enzymes of quinilizidine alkaloid biosynthesis in leaf chloroplasts of Lupinus polyphyllus. *Plant Physiol.* 70 74–77. 10.1104/pp.70.1.74 16662483 PMC1067088

[B89] Wink M. Schimmer O. (1999). Modes of action of defensive secondary metabolites. *Annu. Plant Rev.* 3 17–133.

[B90] Xu W. Zhang Q. Yuan W. Xu F. Aslam M. M. Miao R. (2020). The genome evolution and low-phosphorus adaptation in white lupin. *Nat. Commun.* 11 1–13. 10.1038/s41467-020-14891-z 32103018 PMC7044338

[B91] Yamagishi M. Masuta C. Suzuki M. Netsu O. (2015). Peanut stunt virus-induced gene silencing in white lupin (Lupinus albus). *Plant Biotechnol.* 15:0521.

[B92] Yang H. Lin R. Renshaw D. Li C. Adhikari K. Thomas G. (2010). Development of sequence-specific PCR markers associated with a polygenic controlled trait for marker-assisted selection using a modified selective genotyping strategy: a case study on anthracnose disease resistance in white lupin (Lupinus albus L.). *Mol. Breed.* 25 239–249. 10.1007/s11032-009-9325-4

[B93] Yang T. Nagy I. Mancinotti D. Otterbach S. L. Andersen T. B. Motawia M. S. (2017). Transcript profiling of a bitter variety of narrow-leafed lupin to discover alkaloid biosynthetic genes. *J. Exp. Bot.* 68 5527–5537. 10.1093/jxb/erx362 29155974 PMC5853437

[B94] Zafeiriou I. Polidoros A. N. Baira E. Kasiotis K. M. Machera K. Mylona P. V. (2021). Mediterranean White Lupin Landraces as a Valuable Genetic Reserve for Breeding. *Plants* 10:2403. 10.3390/plants10112403 PMC8619254 34834766

[B95] Zhang N. Huo W. Zhang L. Chen F. Cui D. (2016). Identification of winter-responsive proteins in bread wheat using proteomics analysis and virus-induced gene silencing (VIGS). *Mol. Cell. Proteomics* 15 2954–2969. 10.1074/mcp.m115.057232 27402868 PMC5013310

[B96] Zhao X. Meng Z. Wang Y. Chen W. Sun C. Cui B. (2017). Pollen magnetofection for genetic modification with magnetic nanoparticles as gene carriers. *Nat. Plants* 3:956. 10.1038/s41477-017-0063-z 29180813

